# Morphogen rules: design principles of gradient-mediated embryo patterning

**DOI:** 10.1242/dev.129452

**Published:** 2015-12-01

**Authors:** James Briscoe, Stephen Small

**Affiliations:** 1 The Francis Crick Institute, Mill Hill Laboratory, The Ridgeway, Mill Hill, London NW7 1AA, UK; 2 Department of Biology, New York University, 100 Washington Square East, New York, NY 10003, USA

**Keywords:** Bicoid, *Drosophila* blastoderm, Gene regulatory network, Morphogen interpretation, Sonic hedgehog, Vertebrate neural tube

## Abstract

The *Drosophila* blastoderm and the vertebrate neural tube are archetypal examples of morphogen-patterned tissues that create precise spatial patterns of different cell types. In both tissues, pattern formation is dependent on molecular gradients that emanate from opposite poles. Despite distinct evolutionary origins and differences in time scales, cell biology and molecular players, both tissues exhibit striking similarities in the regulatory systems that establish gene expression patterns that foreshadow the arrangement of cell types. First, signaling gradients establish initial conditions that polarize the tissue, but there is no strict correspondence between specific morphogen thresholds and boundary positions. Second, gradients initiate transcriptional networks that integrate broadly distributed activators and localized repressors to generate patterns of gene expression. Third, the correct positioning of boundaries depends on the temporal and spatial dynamics of the transcriptional networks. These similarities reveal design principles that are likely to be broadly applicable to morphogen-patterned tissues.

## Abstract (precis)

**Summary:** This Review discusses similarities between the mechanisms patterning the *Drosophila* blastoderm and the vertebrate neural tube, suggesting a set of principles for morphogen-patterned tissues.

## Introduction

The importance of gradients in developing embryos and regenerating tissue has long been recognized. From initial proposals more than a century ago, detailed suggestions of the function and nature of embryonic gradients began to take shape (for a review see Rogers and Schier, 2011). These ideas became more concrete in the 1950s and 1960s, with major theoretical contributions from, among others, Alan Turing, Lewis Wolpert and Francis Crick. Turing coined the term ‘morphogen’ to signify biochemical substances that diffuse between cells and generate specific responses at particular concentrations (Turing, 1952). Wolpert introduced the conceptual framework of ‘positional information’ in which developmental pattern formation is dependent on cells interpreting positional values that they have acquired from external signals (Wolpert, 1969). Crick, noticing that pattern specification generally took a few hours and that most developing tissues appeared to be no larger than ∼100 cell diameters, argued, on theoretical grounds, that diffusion was sufficient to establish molecular gradients in tissues (Crick, 1970). Uniting these ideas led to the morphogen theory. This contends that tissue patterning is controlled by a concentration gradient of a morphogen, and that cells acquire positional information by directly measuring the concentration of morphogen to which they are exposed. In this view, specific threshold concentrations establish boundaries of target gene expression, which foreshadow boundaries between cells of different fates.

Although they have evolved over the years to accommodate changing facts and fashions, these ideas have had a profound influence on generations of developmental biologists. The molecular genetics revolution of the 1980s and 1990s led to the identification of several molecules that behave as graded patterning signals (Driever and Nüsslein-Volhard, 1988a; Ferguson and Anderson, 1992; Green and Smith, 1990; Katz et al., 1995; Riddle et al., 1993; Tickle et al., 1985). Subsequent studies revealed that, in most cases, gradients of these molecules are established by dispersion from localized sources and are required for the expression of target genes that are expressed at various distances from the source (reviewed by Rogers and Schier, 2011; Ibañes and Izpisúa Belmonte, 2008; Jeong and McMahon, 2005; Kicheva et al., 2013; Lander, 2013; Lawrence and Struhl, 1996). Recent attention has focused on dissecting the cellular and molecular mechanisms of gradient formation, and advances in imaging and quantitation have contributed fresh insights (Chamberlain et al., 2008; Gregor et al., 2007b; Grimm et al., 2010; He et al., 2008; Kicheva et al., 2007; Little et al., 2011; Zhou et al., 2012). At the same time, complementary studies have aimed to understand how cells respond to graded signals to control differential gene expression (Crauk and Dostatni, 2005; Gregor et al., 2007a; Jiang and Levine, 1993; Robertson, 2014). Finally, a combination of genetics, genomics, misexpression studies, network analysis and mathematical modeling has led to new views of morphogen interpretation (Davidson, 2010; Jaeger et al., 2008; Shilo et al., 2013).

Although gradient formation has been examined in diverse developmental contexts, studies have focused on two examples in particular: Bicoid (Bcd)-mediated patterning of the *Drosophila* blastoderm and Sonic hedgehog (Shh)-mediated patterning of the vertebrate neural tube (Boxes 1 and 2) (for reviews see Alaynick et al., 2011; Dessaud et al., 2008; Jaeger, 2011; Jessell, 2000; Nasiadka et al., 2002; Struhl, 1989). Here, we compare these systems in the context of ideas about gene regulatory networks and dynamical systems theory. This comparison reveals several shared features and suggests that a set of common design principles underpins the patterning of both tissues. These principles form a basis for a revised theory of morphogen-mediated pattern formation. We argue that this theory is likely to be relevant to many tissues and discuss the rationale that might account for this strategy of tissue patterning.

**Box 1. Anterior-posterior (AP) patterning of the *Drosophila* blastoderm**

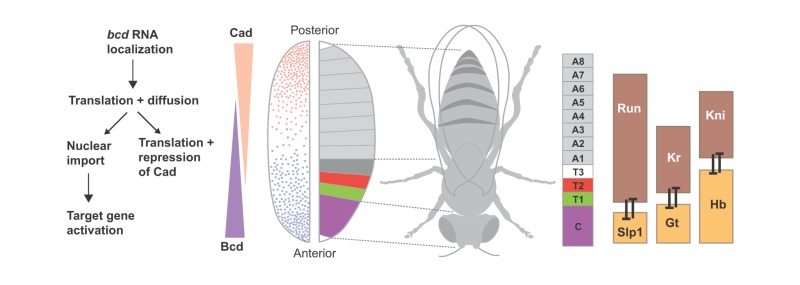

AP patterning of the early *Drosophila* embryo involves maternal gradients of two homeodomain proteins: Bicoid (Bcd) and Caudal (Cad). Bcd protein is translated from a source of mRNA at the anterior pole and diffuses posteriorly through the syncytial blastoderm, forming a long-range AP gradient, with highest levels at the anterior end (Driever and Nusslein-Volhard, 1988a; Little et al., 2011). Complementing the Bcd gradient is an anti-parallel gradient of Cad, which is shaped by Bcd-mediated translational repression (Chan and Struhl, 1997; Niessing et al., 2002). These gradients are initially formed near the cortex of the oocyte, while nuclei divide rapidly in the central region. After ten nuclear division cycles, nuclei migrate to the periphery and import different amounts of Bcd and Cad, depending on their position along the AP axis.

Bcd activates target genes that create boundaries at defined positions along the AP axis, dividing the body plan into regions that will become cephalic (C), thoracic (T1-T3) and abdominal (A1-A8) segments. Bcd target genes include *sloppy-paired 1* (*slp1*), *giant* (*gt*) and *hunchback* (*hb*), which are activated in overlapping domains in anterior regions. *slp1*, *gt* and *hb* encode repressors, which prevent expression of *run*, *Kr* and *kni*, respectively. Mutual repression between these pairs of repressors refines their patterns, creating sharp gene expression boundaries that foreshadow the organization of the body plan (Clyde et al., 2003; Jaeger et al., 2004a; Kraut and Levine, 1991).

**Box 2. Dorsal-ventral (DV) patterning of the vertebrate neural tube**

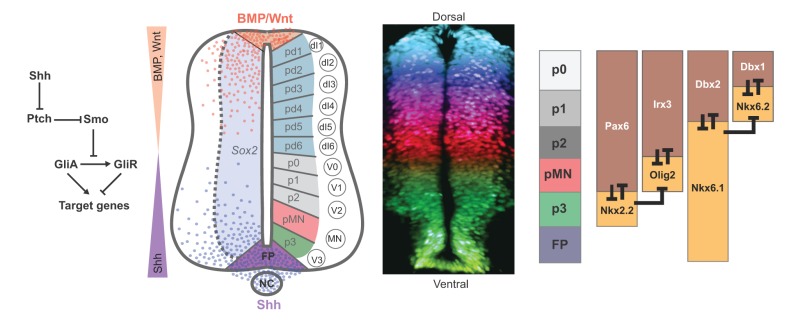

Cell fate specification in the vertebrate neural tube follows a template similar to that in the *Drosophila* blastoderm. Discrete domains of progenitors (p0-p3, pMN, pd1-pd6) are arrayed along the DV axis (Alaynick et al., 2011; Dessaud et al., 2008; Jessell, 2000). Progenitor domain identity is based on the combinatorial expression of a set of TFs and this combinatorial code is necessary and sufficient to specify the neuronal subtypes (V0-V3, MN, dI1-dI6) that each domain generates. The pattern of gene expression is established in a progressive manner in response to opposing gradients of secreted factors: Shh emanating from the ventral pole (NC, notochord); Wnt and BMP signaling dorsally.

Shh binds to the transmembrane receptor Ptch, and this relieves repression on a second transmembrane protein, Smo. Smo activation initiates intracellular signal transduction, culminating in the regulation of Gli family TFs (Briscoe and Thérond, 2013), which are bifunctional transcriptional repressors and activators. In the absence of signal, Gli proteins are either completely degraded or processed to form transcriptional repressors (GliR), whereas Shh signaling inhibits GliR formation and instead activating forms of Gli proteins (GliA) are generated.

In response to the dynamic gradient of Gli activity produced by Shh signaling, the expression of ventral TFs (e.g. Nkx6.1, Olig2, Nkx2.2) are activated, and dorsally expressed TFs (e.g. Pax3, Pax7, Pax6, Msx1, Irx3) are repressed. Binding sites for Gli proteins are associated with genes expressed in the ventral half of the neural tube (Oosterveen et al., 2012; Peterson et al., 2012; Vokes et al., 2007). Many Shh/Gli-regulated genes encode TFs that act as Groucho/TLE-dependent repressors (Muhr et al., 2001). Analogous to the gap proteins, pairs of TFs expressed in adjacent domains cross-repress each other's expression (Briscoe et al., 2000; Vallstedt et al., 2001).

## The *Drosophila* blastoderm and the vertebrate neural tube: distinct but alike

The *Drosophila* blastoderm and the vertebrate neural tube have distinct evolutionary origins that predate the emergence of the bilateria. The signals that act as positional cues in the two tissues are unrelated, the transcription factors (TFs) involved are not orthologous, and the time scales of pattern formation are dissimilar. Establishing the Bcd gradient and the emergence of the gap gene pattern happens within the first ∼2 h of *Drosophila* development (Driever and Nüsslein-Volhard, 1988a; Fowlkes et al., 2008; Surkova et al., 2008). Indeed, gap gene expression is first detected at nuclear cycle 10 and pattern is generally considered fully manifest during nuclear cycle 14, a period of ∼60 min after these genes are initially expressed. In the neural tube, by contrast, the period of patterning varies between species but takes many hours. For example, in chick and mouse embryos the establishment and elaboration of pattern occur over a period of more than 18 h (Dessaud et al., 2007; Jeong and McMahon, 2005). This difference in timing might be directly related to the substantial differences in the cell biology of the two tissues. The *Drosophila* blastoderm is a syncytium with nuclei residing in a shared cytoplasm undergoing synchronized divisions. The absence of cytoplasmic divisions in the blastoderm allows the relatively unfettered movement of TFs between neighboring nuclei, especially during mitosis when the nuclear membranes have broken down. By contrast, the neural tube is a pseudostratified epithelial sheet composed of multiple individual cells proliferating asynchronously. Long-range signaling within the neural tube relies on secreted proteins, which are received by transmembrane receptors and transduced by intracellular signaling pathways.

Despite these differences, there are obvious parallels between the patterning mechanisms in the two tissues. Both are quasi one-dimensional systems with anti-parallel gradients of signaling cues emanating from the two poles of the patterning axis. These cues establish discrete domains expressing sets of TFs that divide the tissues into molecularly distinct blocks of cells arrayed along the patterning axis. In both cases, the combination of TFs expressed in each cell provides the molecular correlate of its position and controls its subsequent development (Boxes 1 and 2). Thus, the problem of pattern formation becomes a question of understanding how the distinct domains of TF expression are generated in an organized and reproducible manner.

**Fig. 1.**
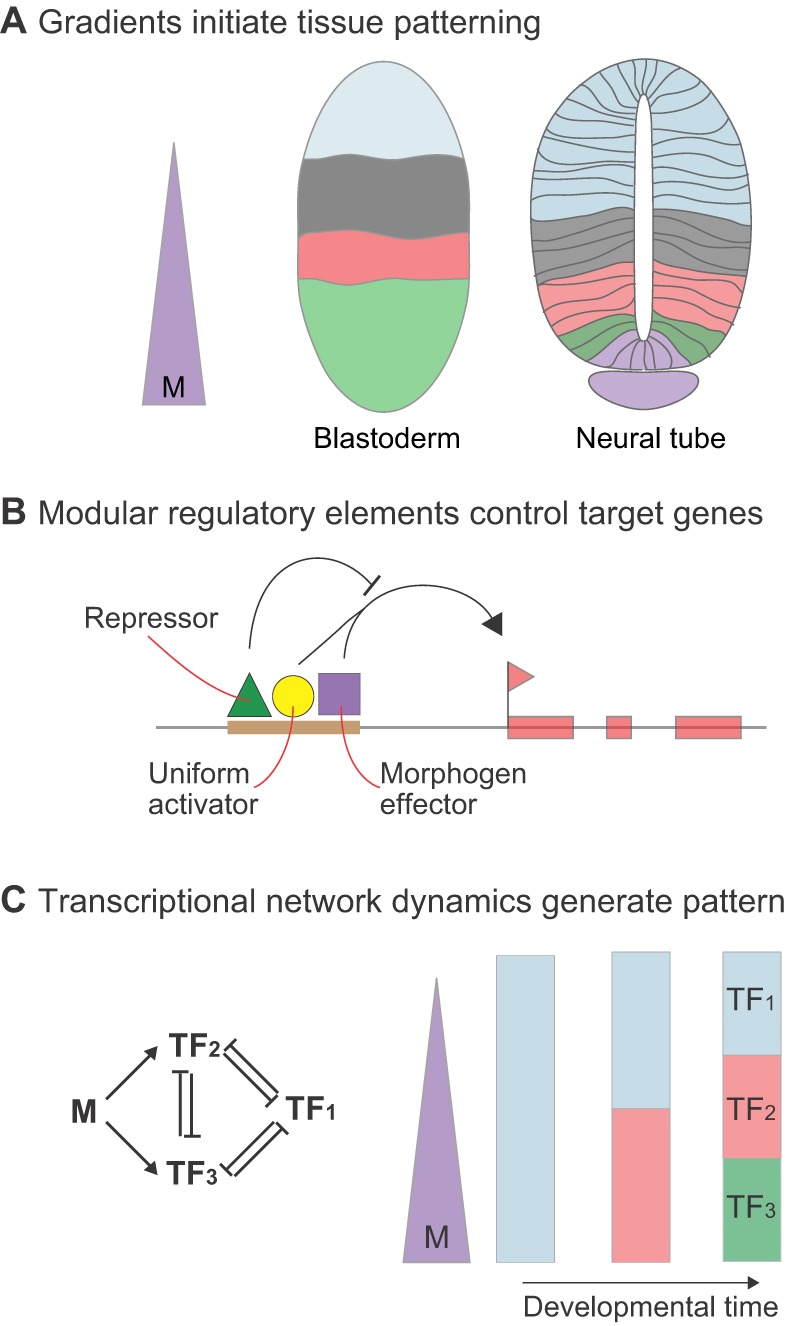
**Design principles of patterning in the *Drosophila* blastoderm and vertebrate neural tube.** (A) Signaling gradients polarize tissues by initiating and orienting gene expression patterns. A morphogen (M, left), Bcd in the case of the blastoderm (center) and Shh for the neural tube (right), forms a gradient. This asymmetry initiates the division of the tissue into domains of gene expression (colored blocks) arrayed along the patterning axis (anterior-posterior in the blastoderm and ventral-dorsal in the neural tube). (B) Patterns of target gene expression are controlled by modular regulatory elements containing binding sites for multiple distinct TFs. These elements integrate transcription inputs from morphogen effectors, uniformly expressed factors, and the transcriptional repressors that comprise the morphogen-regulated transcriptional network. (C) The dynamics of the transcriptional network transform broadly distributed activation and localized repression mechanisms into precisely positioned boundaries of gene expression. This directly links spatial and temporal mechanisms of pattern formation.

Comparing the underlying mechanisms operating in the two tissues supports the idea that there are fundamental similarities in their strategies of pattern formation. Here, we propose an overall design logic to morphogen patterning mechanisms comprising three principles that are shared between the *Drosophila* embryo and vertebrate neural tube patterning systems (Fig. 1). First, we propose that morphogen gradients establish the initial conditions for pattern formation (Fig. 1A). The spatial and temporal input from the gradients determines the state of a transcriptional network by regulating the expression of activating and repressing TFs. Second, target genes are controlled by composite and modular regulatory elements containing binding sites for multiple distinct TFs (Fig. 1B). These elements integrate the transcription inputs to create precise patterns of gene expression. Finally, the dynamics of the transcriptional network transforms the graded input into a precise pattern of gene expression (Fig. 1C), directly linking spatial and temporal mechanisms of pattern formation. Given the separate origins and the molecular and cellular differences between the two systems, these similarities are likely to point towards essential properties of cellular patterning in many – perhaps all – complex tissues.

## Morphogen gradients provide asymmetry but not precise positional information

Genetic and molecular studies indicate that Bcd and Shh act as long-range morphogens within their tissues. In both systems, the absence of the morphogen prevents the formation of some cell types and results in dramatic shifts and expansions of the remaining cell identities into regions normally occupied by the cell types that fail to form. For example, in embryos from mothers lacking Bcd, head and thoracic segments are completely missing and there is a duplication of posterior structures at the anterior end of the embryo (Frohnhöfer et al., 1986). Similarly, in mutant mouse embryos lacking Shh signaling, the cell types found in the dorsal neural tube replace those normally occupying the ventral neural tube (Chiang et al., 1996; Litingtung and Chiang, 2000; Wijgerde et al., 2002). Thus, at the functional level, both Bcd and Shh are involved in two types of activities: the repression of cell fates normally produced at the opposite pole, and the instructive activation of genes required for forming structures where there are high levels of the morphogen.

Several lines of evidence suggest that both Bcd and Shh can function in a concentration-dependent fashion. In the *Drosophila* blastoderm, increasing *bcd* gene copy number shifts the posterior boundaries of Bcd-dependent target genes toward the posterior of the embryo (Driever and Nüsslein-Volhard, 1988b; Struhl et al., 1989). Conversely, changing the number or affinity of Bcd binding sites alters the anterior-posterior (AP) range of *bcd* reporter transgenes: increased binding results in posterior expansion and vice versa (Driever et al., 1989; Simpson-Brose et al., 1994; Struhl et al., 1989). For the neural tube, *ex vivo* experiments using recombinant Shh protein indicate that two- to threefold changes in Shh concentration produce switches in neural progenitor identity (Ericson et al., 1997b; Martí et al., 1995; Roelink et al., 1995). Hence, there is a correlation between ligand concentration and differential gene expression. Comparable changes in neural progenitor identity can also be elicited by modulating the activity level of intracellular Gli – the transcriptional effector of Shh signaling (Stamataki et al., 2005). Together, these data appear to support the conventional view of a morphogen in which boundaries of gene expression correspond to specific thresholds of morphogen activity, implying that the concentration of a patterning signal is a direct measure of positional information.

**Fig. 2.**
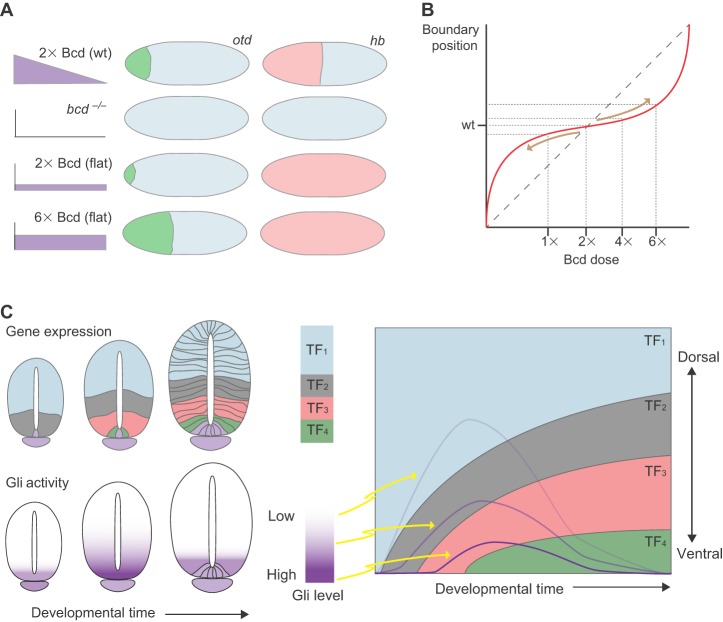
**Target gene expression boundaries do not correlate with simple concentration thresholds.** (A) Boundaries of the Bcd target genes *otd* and *hb* are set at specific positions in wild-type (wt; 2× Bcd) embryos. Neither gene is expressed in embryos laid by *bcd* mutant (*bcd*^−/−^) females. When the Bcd gradient is flattened by genetic manipulation, the expression of *otd* and *hb* is restored but *otd* expression shows a sharp boundary that shifts posteriorly when *bcd* copy number is increased from two to six. By contrast, *hb* is expressed throughout the embryo in response to the flattened Bcd gradient. In embryos with flattened gradients, both *otd* and *hb* can be activated by lower concentrations of Bcd than those associated with their boundary positions in wild-type embryos. (B) *Drosophila* embryos with altered Bcd dosage (*x*-axis) show shifts in target gene boundary positions (*y*-axis), but these (red line) are smaller than predicted by a linear relationship between Bcd dose and boundary position (dashed line). (C) In the neural tube, progenitor identities (upper images) are established sequentially, with identities corresponding to higher morphogen concentrations appearing after longer periods of signaling. As a consequence, ventral progenitors exposed to high concentrations of Shh transiently adopt a gene expression profile associated with fates induced by lower concentrations. Measurements of Gli activity (bottom images, purple gradient) indicate that the amplitude and range of the gradient change over time. The level of Gli activity initially increases before decreasing, creating an adapting response. Correlating Gli activity levels with individual expression boundaries indicates that a boundary of gene expression is associated with different levels of Gli activity at different developmental times.

However, findings from both the blastoderm and neural tube challenge the strict relationship between signal concentration and positional identity. In embryos in which the Bcd gradient has been flattened by genetic manipulation, several target genes continue to form well-defined boundaries that are shifted in position but nonetheless correctly ordered along the patterning axis (Fig. 2A,B) (Chen et al., 2012; Löhr et al., 2009; Ochoa-Espinosa et al., 2009). Moreover, in these embryos the boundaries of target genes are associated with lower concentrations of Bcd than in wild-type embryos, suggesting that Bcd is in excess at every position within the wild-type gradient (Ochoa-Espinosa et al., 2009). Finally, during the process of pattern formation, the position of gap gene expression boundaries changes relative to Bcd levels, directly demonstrating the lack of a simple relationship between morphogen concentration and threshold responses (Jaeger et al., 2004b).

Absolute levels of morphogen also do not appear to dictate gene expression in the neural tube. Measurements of Gli activity *in vivo* reveal temporal changes in the levels of signaling at individual expression boundaries (Fig. 2C) (Balaskas et al., 2012; Junker et al., 2014). The identities of neural progenitor domains are established sequentially, with identities corresponding to higher morphogen concentrations requiring longer periods of signaling (Dessaud et al., 2007, 2010; Jeong and McMahon, 2005). As a consequence, ventral progenitors exposed to high concentrations of Shh transiently adopt a gene expression profile associated with fates induced by lower concentrations. The result is that gene expression boundaries are associated with different levels of signaling over time (Balaskas et al., 2012; Junker et al., 2014). This suggests a dynamic system in which the duration, as well as the level of morphogen signaling, is critical for neural progenitor patterning. Moreover, disruptions of patterning and loss of ventral cell types observed in *Shh* mutant embryos can be recovered, to a significant extent, in double-mutant embryos lacking Shh and Gli3, the Gli family member that functions predominantly as a transcriptional repressor (Litingtung and Chiang, 2000; Persson et al., 2002). Thus, similar to Bcd, absolute levels of Shh/Gli activity do not appear to be sufficient to determine gene expression patterns.

Nevertheless, the Bcd and Shh gradients are essential for pattern formation (Briscoe et al., 2001; Driever et al., 1990; Frohnhöfer et al., 1986; Staller et al., 2015a; Wijgerde et al., 2002). Reconciling these apparently contradictory conclusions leads to the view that gradients provide an initial polarization that biases positional identity, but absolute levels of signal do not directly imprint a spatial metric to the developing cells. In this view, gradients of quite different amplitudes could still function in target gene patterning. There is some experimental precedent for this idea. For example, the survival of embryos laid by *bcd* heterozygotes, which contain only half the maximal amount of Bcd present in wild-type embryos, suggests that the critical thresholds within the gradient (if any) are confined to the lower half of the concentration range. In addition, genetic and transgenic techniques have been used to generate embryos with Bcd gradients that differ by up to fivefold in their maximal concentrations (Fig. 2B) (Driever and Nüsslein-Volhard, 1988a; Liu et al., 2013; Struhl et al., 1989), and all these embryos survive to fertile adulthood in laboratory conditions.

The ability to genetically change the amplitude of the Bcd gradient led to critical tests of the hypothesis that target gene boundaries are positioned by threshold-dependent mechanisms. If boundaries are positioned by specific thresholds, it should be possible to predict how far each boundary shifts when the Bcd profile is changed. Previous studies showed that the boundary shifts in such experiments are less dramatic than predicted by the simple morphogen model (Gao and Finkelstein, 1998; Gao et al., 1996; Houchmandzadeh et al., 2002). More recent quantitative analysis showed that boundary positioning is a time-dependent process (Liu et al., 2013). When target genes are first expressed, boundaries shift to positions very close to those predicted by the morphogen model. However, within minutes, these initial shifts are reduced in degree, back toward their positions in wild-type embryos, which is consistent with the previous studies. These results suggest the existence of mechanisms that buffer fluctuations in gradient amplitude and shape (see below).

## Morphogens function with transcriptional networks to refine gene expression boundaries

If specific morphogen concentration thresholds are not crucial for patterning, what explains the patterns of gene expression? Insight has come from bioinformatic and genomic analyses of cis-regulatory elements (CREs) associated with differentially expressed genes. DNA binding and chromatin immunoprecipitation assays provide evidence that Bcd and Gli proteins directly activate the expression of many target genes expressed in regions that coincide with the spatial extent of the morphogen gradient. For example, Bcd binding sites are observed in regulatory elements of more than 50 different target genes, most of which are expressed in anterior and central regions of the embryo (Chen et al., 2012; Ochoa-Espinosa et al., 2005; Segal et al., 2008). Similarly, genes induced in the ventral half of the neural tube are associated with Gli binding sites (Oosterveen et al., 2012, 2013; Peterson et al., 2012; Vokes et al., 2007).

**Fig. 3.**
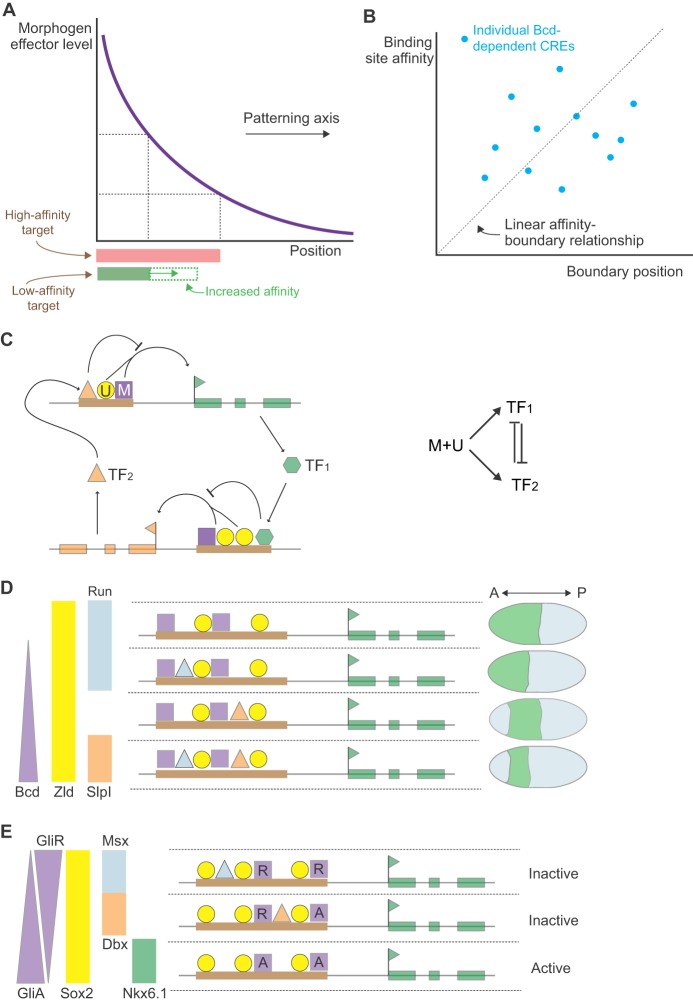
**The cis-regulatory mechanisms controlling gene expression.** (A) A simple mechanism of morphogen gene regulation is that target gene boundary position is determined directly by the binding affinity of CREs for the morphogen effector. The affinity of the CRE thus determines the amount of activated morphogen effector (purple) bound and is predicted to correlate with the extent of gene expression. High-affinity sites (red) produce long-range induction, whereas low-affinity binding sites (green) result in more restricted gene induction. Increasing the affinity of these binding sites (dotted green) would expand the range of gene induction. (B) There is a lack of correlation between boundary positions (*x*-axis) of a set of Bcd target genes (blue points) and the affinity of Bcd binding sites (*y*-axis) in the CREs associated with the target genes. (C) The CREs of target genes within the patterning network combine three classes of transcriptional inputs. The morphogen effectors (M, purple) act broadly to regulate many target genes along their patterning axis. Input from uniformly expressed factors (U, yellow) change the sensitivity of individual target genes to morphogen input. Repressive input from pd-TFs (TF_1_ and TF_2_) regulated by the network inhibit the positive activity of the morphogen and uniform factors. The integration of these inputs produces the regulatory logic of the transcriptional network. (D) Patterning by combinatorial binding in the blastoderm. The expression patterns of two activators (Bcd and Zld) and two repressors (Slp1 and Run) are shown (left). Hypothetical CREs are also shown (center) with their predicted expression patterns (right). The top construct contains only activation inputs and is expressed throughout the anterior embryo. The addition of repressor sites restricts activation to specific regions and positions the boundaries of gene expression. (E) Nkx6.1 is expressed in the ventral third of the neural tube. An Nkx6.1 CRE recapitulates this expression and contains a combination of binding sites for Gli, Sox2 and the pd-TFs Dbx and Msx. In the ventral neural tube, the absence of repressor forms of Gli and the lack of Dbx and Msx expression allows Sox2 proteins to activate the CRE. Dorsal to this, the presence of Gli repressors and Dbx or Msx blocks the activity of the CRE. GliA and GliR, activator and repressor forms of Gli.

One mechanism, initially proposed to explain morphogen activity (Driever et al., 1989), is that target gene boundary position is determined in a straightforward manner by the binding sensitivity of CREs for the morphogen effector (Fig. 3A) (Driever et al., 1989; Struhl et al., 1989). In this ‘binding affinity’ model, CREs that contain binding sites with low affinity for the morphogen effector would be bound (and active) only in regions containing high morphogen levels, whereas CREs with high-affinity binding sites would also be bound in regions containing lower levels of morphogen. However, the analysis of CREs associated with sets of Bcd and Shh target genes does not support this. For example, the boundary positions of a set of Bcd target genes do not correlate with the affinity or number of Bcd binding sites in their associated CREs (Fig. 3B) (Ochoa-Espinosa et al., 2005). Similarly, Shh target genes in the neural tube lack the expected correlation between the affinity of Gli binding sites and the range of gene induction (Oosterveen et al., 2012; Peterson et al., 2012). Indeed, the only noticeable trend in these datasets was that more ventrally restricted genes appear to contain high-affinity binding sites. This is opposite to the predictions of the binding affinity model. It should be noted, however, that this model is founded on the assumption that the morphogen effector is latent in the absence of signal and converted to a transactivator by the morphogen. In the case of Shh signaling, Gli family members bind to the same regulatory elements as their Shh-activated counterparts but act as transcriptional repressors (see Box 2). Nevertheless, these data argue against the idea that a simple hierarchy of differential binding sensitivity determines target gene expression boundaries.

In addition to binding morphogen effectors, the CREs controlling spatial and temporal patterning bind multiple TFs (Fig. 3C). Some of these are ubiquitously expressed transcriptional activators that play important roles in activating gene expression. For example, in the blastoderm the uniformly expressed TF Zelda (Zld; Vielfaltig – FlyBase) is necessary for correct gap gene pattern (Liang et al., 2008; Xu et al., 2014). Zld binds to the regulatory elements of many of the gap genes, and altering these interactions affects the binding of Bcd to DNA and Bcd-dependent expression patterns. The differential binding of Zld to a subset of target genes provides a mechanism by which the sensitivity of target genes to a morphogen effector can be modified independently of the effector itself (Kanodia et al., 2012). In the neural tube, SoxB1 family TFs (Sox1-3), which are expressed in all neural progenitors, appear to play a Zld-like role in modulating Shh signaling (Bergsland et al., 2011; Oosterveen et al., 2012; Peterson et al., 2012). Binding sites for SoxB1 proteins have been identified and functionally implicated in regulatory elements associated with neural progenitor TFs. Thus, the number, affinity or arrangement of SoxB1 binding sites within a given element could influence its response to Shh-Gli input.

Also found in target gene CREs are binding sites for TFs that are under the transcriptional control of Bcd and Gli activity (Fig. 3C). We will refer to these TFs as pattern-determining TFs (pd-TFs). A combination of developmental genetics and quantitative approaches indicates that pd-TFs form transcriptional networks that play central roles in morphogen interpretation. The pd-TFs function predominantly as transcriptional repressors and, in both the *Drosophila* embryo and the vertebrate neural tube, pairs of pd-TFs expressed in neighboring domains cross-repress each other (see Boxes 1 and 2 for details) (Briscoe et al., 2000; Clyde et al., 2003; Ericson et al., 1997b; Kraut and Levine, 1991; Vallstedt et al., 2001). This cross-regulation creates bistable switches that stabilize and sharpen gene expression domains, culminating in all-or-nothing gene expression boundaries between cells containing different repressors. The cross-repressive interactions also contribute to the positioning of gene expression boundaries along the patterning axis. Mutations in one or more pd-TF(s) cause predictable shifts in the pattern of expression of the remaining pd-TFs without affecting the morphogen gradients themselves (Fig. 3D). This further dissociates positional identity from the absolute level of morphogen signal. For example, the gap gene *hunchback* (*hb*) is expressed in the anterior half of the fly embryo and is responsible for restricting the abdominal gap gene *knirps* (*kni*) to posterior regions (Clyde et al., 2003; Pankratz et al., 1992; Yu and Small, 2008). In mutants lacking *hb*, the *kni* expression domain expands anteriorly into regions normally occupied by *hb*. Two other mutually repressive pairs, Gt and Kruppel (Kr), and Slp1 and Run, also form bistable switches that create additional boundaries in more anterior regions (Box 1) (Andrioli et al., 2004; Wu et al., 1998). In the neural tube, the TF Nkx2.2 is expressed in a domain that ventrally abuts progenitors expressing Pax6; in embryos lacking Pax6, Nkx2.2 expression expands dorsally and, consequently, the neuronal subtypes produced from these progenitors also increase (Ericson et al., 1997a). The pd-TFs Olig2 and Irx3, as well as Nkx6.1 and Dbx2, also form bistable switches that demarcate additional boundaries in the ventral neural tube (Box 2) (Novitch et al., 2001; Sander et al., 2000; Vallstedt et al., 2001). Taken together therefore, these findings suggest that the transcriptional repressors downstream of the morphogen create a transcriptional network that ensures cells select a single discrete identity and position the boundaries between distinct regions along the patterning axis.

Together, the analyses of CREs suggest a strategy for reading a morphogen gradient that involves the combined activity of three classes of transcriptional inputs (Chen et al., 2012; Oosterveen et al., 2012; Xu et al., 2014). First, the morphogen effectors act broadly to regulate many target genes along their patterning axes. Second, input from uniformly expressed factors changes the sensitivity of individual target genes to morphogen input. Finally, inputs from cross-repressing pd-TFs, which are themselves differentially regulated by the network, generate switches in gene expression that create discrete boundaries and determine the positions of these expression boundaries (Fig. 3D). For example, regulatory sequences associated with the Bcd target gene *orthodentical* (*otd*; *ocelliless* – FlyBase) contain clusters of binding sites for Bcd, Zld and for Hb, which functions as an activating co-factor with Bcd through a feedforward loop (Gao and Finkelstein, 1998; Ochoa-Espinosa et al., 2005; Simpson-Brose et al., 1994; Xu et al., 2014). Binding sites for all three proteins may contribute to activation of *otd* expression. The *otd* regulatory sequences also contain binding sites for the repressor Run and the maternally expressed repressor Capicua, which are crucial for restricting Otd expression to presumptive head regions of the embryo (Chen et al., 2012; Löhr et al., 2009). Similarly, in the neural tube, detailed analysis of a regulatory element associated with Nkx6.1 identified a combination of binding sites for SoxB, Gli and homeodomain TFs (Fig. 3E) (Oosterveen et al., 2012). Each of these appears to contribute to the regulation of Nkx6.1, with different homeodomain proteins repressing Nkx6.1 in different territories along the patterning axis of the neural tube. Thus, the pattern of gene expression is not governed solely by the concentration of morphogen effector, but instead is controlled by a combination of morphogen effector levels, uniformly expressed factors and the TFs regulated by the morphogen. It is the combination of inputs, and not the absolute level of morphogen effector, that provides the correlate of positional information in the tissue.

## Target gene CREs integrate multiple transcriptional inputs

Although detailed molecular mechanisms of how individual CREs control transcription remain to be fully delineated, the analysis of several CREs associated with blastoderm expressed genes has provided some clues. Activation seems to be combinatorial, involving more than one activator protein, in all cases examined so far. This might be the result of protein-protein interactions: for example, the uniformly expressed factor Zld promotes the binding of Bcd, suggesting a cooperative mechanism (Xu et al., 2014). Alternatively, or in addition, Zld and Bcd may function independently, in an additive fashion. SoxB TFs also seem to function in a similar manner with Gli proteins to contribute to neural gene regulation (Bergsland et al., 2011; Oosterveen et al., 2012, 2013; Peterson et al., 2012).

In general, transcriptional activators appear to function over significant distances, with CREs often sited many kilobases from the transcription start site of the genes they regulate (Davidson, 2010; Kvon et al., 2014). By contrast, repressors appear to act locally, at the regulatory element to which they bind, to suppress the activators bound to the same CRE (Gray et al., 1994; Small et al., 1993). In some cases, the binding sites for activators and repressors either overlap or are closely linked, and hence competition for binding is an important mechanism (Small et al., 1992). Alternatively, repressors can work over short distances within a regulatory element to inhibit activators bound within ∼200 bp (Gray and Levine, 1996). Thus, the binding of a repressor to a regulatory element could suppress positive transcriptional activity either by displacing activators or quenching the activity of the bound activators.

The available data suggest that the CREs associated with target genes integrate multiple inputs to ‘compute’ how each associated target gene is regulated (Fig. 3C-E) (Segal et al., 2008; Wilczynski et al., 2012). A consequence of this mechanism is that none of the individual TFs functions as a master regulator, which is consistent with the lack of a strict correlation between the binding affinity of morphogen effectors and the response of individual genes. For each CRE, it is the combination of positive and negative inputs that determines how the associated gene responds. Thus, CREs link the combinatorial regulatory logic of the network with its molecular implementation in the genome. This suggests a flexible but robust means to establish and evolve patterns of gene expression. For example, moving repressor binding sites various distances from activator sites might allow alterations in the strength of repression to fine-tune position boundaries while still generating the bistability necessary for boundary formation (Gray et al., 1994; Hewitt et al., 1999).

In both the *Drosophila* blastoderm and the vertebrate neural tube, multiple regulatory elements are associated with many of the patterning genes. For example, the Bcd target gene *hb* contains two distinct CREs, harboring clusters of Bcd sites. These direct very similar patterns of expression in the anterior half of the embryo (Perry et al., 2011). This supports the idea of ‘shadow enhancers’, in which the principal CRE (or the first identified regulatory element) is ‘shadowed’ by additional CREs with similar activity (Barolo, 2012; Hong et al., 2008a; Perry et al., 2010). An analogous phenomenon also appears to operate in the neural tube. Analyses of chromatin binding identified two or more discrete regions co-bound by Gli1/Sox2, coinciding with blocks of sequence conservation, around many of the genes encoding pd-TFs activated by Shh signaling in the ventral neural tube (Oosterveen et al., 2012, 2013; Peterson et al., 2012). Functional assays confirmed the neural-specific CRE activity for many of these regions. In the majority of cases, different CREs from the same gene had similar, albeit not identical, patterns of activity (Perry et al., 2011). The similarity in activity despite differences in the composition of the elements indicates that there are multiple ways in which the same pattern of gene expression can be produced.

Several possibilities have been put forward to explain why genes contain multiple CREs with apparently similar activities (Barolo, 2012; Hong et al., 2008a; Perry et al., 2010). One possibility is that different CREs have distinct functions in the interpretation of the graded input. Although the collective analyses of regulatory elements has failed to find a clear correlation between the binding strength for the morphogen effector and the pattern of activity of the element (Ochoa-Espinosa et al., 2005), it is possible that a correlation does exist for a subset of elements. The activity of these CREs would then be directly instructed by the morphogen gradient. These could play directorial roles by establishing appropriate patterns of key pd-TFs in the network that then drive pattern formation. In this view, all other CREs in the system would require morphogen input for activation, but this would be permissive, and the input from already patterned repressors would make the contributions to target gene boundary positioning. Nevertheless the coordinated shifts in gene expression that result from the deletion of individual repressors in the transcriptional network suggest that the network dominates the graded input and is the main driver of pattern formation.

Apparently redundant regulatory elements could also contribute to the robustness of pattern formation (Perry et al., 2010). Advances in imaging techniques and the increasing resolution of data generated by these approaches are beginning to reveal that transcription is a noisy, bursty process (Darzacq et al., 2007; Elowitz et al., 2002; Garcia et al., 2013). Thus, it is likely to be difficult to control a single element in a precise way. Combining multiple independent CREs might average fluctuations in gene expression that result from regulation by a single enhancer and increase the frequency of expressing nuclei (Perry et al., 2012). This could increase the robustness and reliability of pattern formation in the face of environmental stresses, such as the varying temperatures that developing embryos are exposed to in the wild. Alternatively, or in addition, the multiple CREs could function to fine-tune the spatial or temporal pattern of gene expression (Perry et al., 2011; [Bibr DEV129452C133][Bibr DEV129452C134]). It is also possible that multiple CREs combine to produce additive or synergistic interactions to ensure rapid changes in gene induction or boost expression levels. Finally, the presence of multiple semi-redundant elements might offer evolvability by weakening the selective constraints on individual elements and allowing some evolutionary drift (Hong et al., 2008a). Nevertheless, the idea that multiple CREs increase robustness and evolvability must take account of the apparently distinct mechanisms of the long-range function of activators and the local action of repressor TFs. In this case, the absence of repressor binding to one CRE would result in inappropriate gene expression even if the other CREs associated with the gene remained inhibited.

## Integrating graded positional information with patterning networks: insights from mathematical modeling

The experimental approaches outlined above have identified many of the molecular components of the patterning network and provided insight into the regulatory architecture that connects them, but they do not offer a detailed explanation for how the spatial pattern forms in each of the tissues. Mathematical models based on the experimental data, which describe the dynamics of the transcriptional networks, shed light on this issue.

**Fig. 4.**
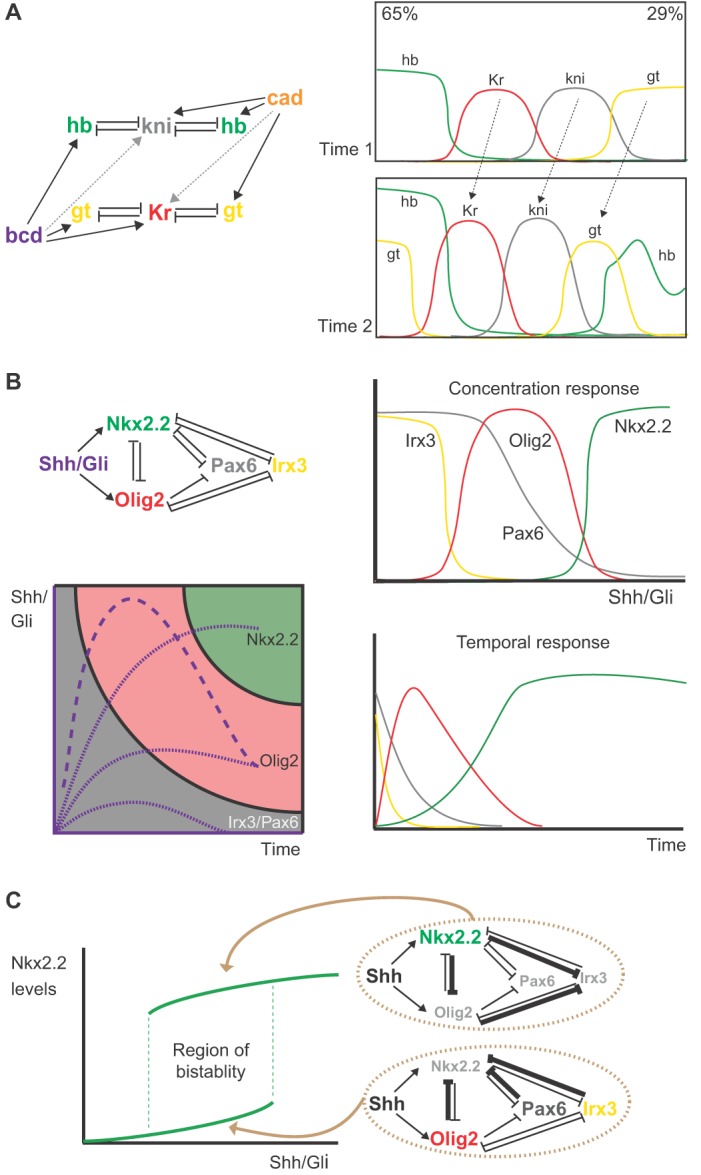
**The dynamics of the transcriptional network generate pattern.** (A) A mathematical model of the gap gene network recapitulates the temporal-spatial pattern along the AP axis of the blastoderm. Cross-regulatory interactions between gap gene pairs establish the initial patterns of pd-TF expression in middle regions of the embryo (65% to 29% embryo length) (time 1). Asymmetries in the strength of cross-repression between gap genes means that posterior gap genes dominate over their more anterior partners. As development proceeds (time 2), this leads to the gradual sharpening and an anterior shift of the entire gap gene expression pattern. (B) A transcriptional circuit comprising four pd-TFs (Nkx2.2, Olig2, Irx3 and Pax6) linked by a series of cross-repressions determines the response of these genes to Shh-Gli signaling and positions the two progenitor domain boundaries that they define. A mathematical model of the circuit recapitulates the pattern and temporal sequence of gene expression observed in neural progenitors: Olig2 expression is induced in ventral neural progenitors before Nkx2.2; Nkx2.2 induction represses Olig2, resulting in an overall dorsal shift in pattern *in vivo*. A phase portrait based on the mathematical model illustrates the connections between the levels or durations of signal. Compared with Olig2, the induction of Nkx2.2 requires higher levels and longer durations of Shh-Gli activity. The dynamics of Shh signaling at three different positions in the neural tube are indicated with dotted purple lines. The portrait also illustrates that transient high levels of signaling at early times (purple dashed line) are not sufficient to switch from Olig2 to Nkx2.2, provided that this level of signaling is not sustained. (C) The transcriptional circuit produces hysteresis. Nkx2.2 induction by Shh-Gli signaling requires the repression of Pax6 and Olig2; this necessitates high levels of Gli activity (bottom green line). Once induced, Nkx2.2 inhibits Pax6 and Olig2 expression, thereby allowing Nkx2.2 expression to be sustained at lower levels of Shh-Gli signaling (top green line). This might explain how gene expression is maintained as Shh-Gli activity decreases below inducing levels.

A dynamical model of the gap gene network, based on quantitative data from embryos, was sufficient to simulate the establishment of AP pattern and revealed that cross-regulatory interactions between gap gene pairs are responsible for the observed Bcd-independent shifts in the expression of these genes (Fig. 4A) (Jaeger et al., 2004b; Manu et al., 2009a,b). Key to this behavior is that the strength of cross-repression between gap genes is asymmetric, with posterior gap genes dominating over their more anterior partners. This leads to a cascade of asymmetric feedback that sharpens and shifts the entire gap gene expression pattern anteriorly as development proceeds.

A similar dynamical mechanism appears to operate in the neural tube network (Fig. 4B). A transcriptional circuit comprising four Shh-regulated pd-TFs (Nkx2.2, Olig2, Irx3 and Pax6) linked by a series of cross-repressions has been explored in detail (Balaskas et al., 2012; Cohen et al., 2014; Panovska-Griffiths et al., 2013). The strengths of cross-repressive interactions between the pd-TFs appear to determine the response of these genes to Shh-Gli signaling and, consequently, the positioning of the two progenitor domain boundaries that they define. The model accurately predicts the temporal sequence of gene expression observed in neural progenitors. For instance, both *in vivo* and *ex vivo*, primary neural cells exposed to a fixed concentration of recombinant Shh induce Olig2 expression in ventral neural progenitors before inducing Nkx2.2 (Dessaud et al., 2007; Jeong and McMahon, 2005). Subsequently, Olig2 is repressed as Nkx2.2 is induced, resulting in an overall dorsal shift in pattern *in vivo*. This behavior is recapitulated in mathematical models. Surprisingly, the models predict that the transcription network can generate the differential temporal and spatial behavior of Nkx2.2 and Olig2 even if both genes receive identical inputs from the morphogen. This leads to the conclusion that the differential responses of the patterning genes to different levels and periods of morphogen signaling are a consequence of the regulatory logic of the transcriptional network. Thus, the dynamics of the transcriptional network are responsible for both spatial and temporal patterns of gene expression.

The same models also help to explain other experimentally observed behavior. As mentioned above, gene expression shifts caused by altering Bcd levels are less severe than predicted. A detailed mathematical simulation of the gap gene system indicated that the regulatory interactions between gap genes mean that the gene expression profile adopted by each nucleus is stable against perturbations within certain ranges (Manu et al., 2009a,b). This suggests that cross-regulation between gap genes provides some error correction downstream of the Bcd gradient that improves the precision and reliability of gap gene expression boundaries. Consistent with this, embryos mutant for either of two gap genes, *Kr* and *kni*, have higher variability in the position of the remaining domain boundaries than wild-type embryos (Surkova et al., 2013). In the neural tube, embryos lacking the repressor Gli3 display transiently increased levels of Gli activity (Balaskas et al., 2012). Despite this increased signaling, the position of the *Nkx2.2* and other gene expression boundaries in the ventral neural tube appear unchanged (Persson et al., 2002). Inspection of a mathematical model of the transcriptional network suggested that cross-repressive interactions between Pax6 and Nkx2.2 could explain this insensitivity to the temporary increase in Gli activity. In line with this, a double mutant lacking both Gli3 and Pax6 displayed a markedly increased shift in the border of Nkx2.2 expression (Balaskas et al., 2012). This suggests that the network makes cells insensitive to transient fluctuations in signaling levels and provides a means for cells to effectively average morphogen signaling over time.

The tools and concepts from dynamical systems theory provide a convenient way to appreciate and visualize these ideas (Jaeger and Monk, 2014; Jaeger et al., 2008; Strogatz, 2014). For example, ‘phase portraits’ (Fig. 4B) based on mathematical models of the networks can be used to illustrate the connection between how a system responds to different levels or durations of signal. In the case of the transcriptional network in the ventral neural tube, such an analysis indicates that, compared with Olig2, the induction of Nkx2.2 requires higher levels and longer durations of Shh-Gli activity (Fig. 4B). In addition, the portrait illustrates that a transient increase in signaling at early times, even if it is above the threshold necessary for Nkx2.2 induction, is not sufficient to switch from Olig2 to Nkx2.2 induction. This emphasizes that there are not separate mechanisms for spatial and temporal patterning: both are the product of the transcriptional network.

An additional layer of patterning complexity is found in the neural tube, where the levels of morphogen signaling activity change over time (Fig. 2C) (Balaskas et al., 2012; Chamberlain et al., 2008; Junker et al., 2014). As a consequence, there is no constant relationship between position and the level of signaling. In the ventral neural tube Shh protein production increases during development, resulting in an increasing maximum concentration of Shh at the ventral pole of the neural tube (Chamberlain et al., 2008; Cohen et al., 2015). Downstream Gli transcriptional activity also initially increases but then decreases despite the increasing concentrations of Shh. These adapting dynamics have been proposed to arise from a combination of three mechanisms: negative feedback induced by Shh signaling, transcriptional downregulation of Gli gene expression, and the differential stability of active and inactive Gli isoforms (Cohen et al., 2015; Junker et al., 2014). Irrespective of the relative contributions of each of these mechanisms, the result is that the level of Gli activity associated with a particular progenitor identity is higher than the level of Gli activity in these cells at a later time. Models of the neural tube transcriptional network suggest that mutual repression between pairs of TFs could provide an explanation for how gene expression is maintained as Shh signaling decreases below the inducing levels. In dynamical systems terminology, the network produces a phenomenon known as ‘hysteresis’ (Strogatz, 2014). This is a property of multistable systems in which the state of the system is dependent on the history of inputs it has received, as well as the current input. In the case of the neural tube, the induction of Nkx2.2 by Shh signaling requires the repression of Pax6 and Olig2 but, once induced, Nkx2.2 inhibits the expression of these genes thereby allowing Nkx2.2 expression to be sustained at lower levels of Shh-Gli signaling (Fig. 4C) (Balaskas et al., 2012). In essence, the induction of Nkx2.2 and the repression of Pax6 and Olig2 act as a memory of the past input of Shh signaling. Hence, just as the temporal sequence of gene expression can be explained by the dynamics of the transcriptional network so too can the maintenance of gene expression as a tissue is elaborated (Dessaud et al., 2010; Su et al., 2012). This does not exclude the possibility that alternative molecular mechanisms, such as chromatin modifications, also play a role in stabilizing pattern; however, the structure and dynamics of the transcriptional network provide a means to accomplish this without the need for additional layers of regulation.

The focus on signaling dynamics raises the possibility that gradients are interpreted prior to reaching steady state. Theoretical work suggests that this can reduce the effects of fluctuations and thereby increase the precision of spatial boundaries (Bergmann et al., 2007; Saunders and Howard, 2009; Tamari and Barkai, 2012). In addition, the kinetics of target gene responses could be exploited to control differential gene responses: target genes with a high transcription rate are rapidly expressed to produce an early onset and long-range pattern, whereas genes with lower transcription rates produce shorter-range responses. Such a mechanism has been proposed for Nodal signaling during mesendoderm induction in zebrafish (Dubrulle et al., 2015). The consideration of signaling dynamics also leads to the idea that cells use the temporal derivative or integral of the signal to pattern a tissue. Behavior consistent with this has been suggested for TGFβ (Sorre et al., 2014) and Dpp (Wartlick et al., 2011). This could result in more accurate patterning than that achieved by mechanisms based on simply interpreting absolute morphogen concentration (Richards and Saunders, 2015). Mechanistically, the way cells ‘calculate’ a derivative or integral would probably rely on the downstream transcriptional network. In the case of the neural tube, the transcriptional network could be described as a system that uses the integral of Shh signaling to define gene expression patterns.

As mentioned above, there are marked differences in the time scale over which patterning takes place in the blastoderm and neural tube. What causes this difference in time scales is unclear. Several features of the two tissues might contribute. In the blastoderm, the syncytial structure allows gradients of TFs to form promptly and directly in the shared cytoplasm. In the cellularized neural tube, however, morphogen signaling relies on extracellular gradients transduced through intracellular cascades. It is notable that, in the case of Shh signaling, transduction is unlikely to be rapid because it relies on the degradation of repressor isoforms of the Gli proteins and the gradual accumulation of newly synthesized Gli proteins that can be converted into activated isoforms (Briscoe and Thérond, 2013). However, in addition to these differences in the kinetics of the patterning cues, properties of the repressor proteins might also contribute to the different time scales (Cohen et al., 2015; Humke et al., 2010). For example, gap proteins have relatively short half-lives and are mostly degraded by the onset of gastrulation (Kraut and Levine, 1991; Pisarev et al., 2009), which would allow the transcriptional network to approach its steady state more rapidly than could be achieved if the gap genes were long lived. The half-lives of the neural tube TFs have not been measured but their stability might contribute to the rate of patterning, and it is possible that modulating these half-lives provides a mechanism to alter the speed of pattern formation in distinct species.

## Anti-parallel gradients and pattern scaling

Another common feature of the two developmental systems is that both involve anti-parallel patterning cues emanating from the opposite poles of the patterning axis. The anterior gradient of Bcd in the blastoderm is complemented by a gradient of the TF Caudal (Cad) emanating from the posterior pole (Mlodzik and Gehring, 1987). In the neural tube, gradients of BMP and Wnt from the dorsal pole complement the ventral Shh gradient (Barth et al., 1999; Jessell, 2000; Muroyama et al., 2002; Nguyen et al., 2000). In both tissues, the anti-parallel gradients have opposing activities. Bcd activates anterior gap gene expression, whereas Cad promotes the expression of more posterior gap genes (Rivera-Pomar et al., 1995). In addition, Bcd represses Cad translation in anterior regions of the embryo via direct binding to *cad* RNA (Chan and Struhl, 1997; Niessing et al., 2002). This creates a gradient of Cad protein that is shaped directly by the Bcd protein gradient. Removal of Bcd expands the Cad expression domain into anterior regions, which probably contributes to (but is not sufficient for) the posteriorization of this region in *bcd* mutants. In the neural tube, the activation of ventral gene expression by Shh signaling is opposed by BMP and Wnt (Alvarez-Medina et al., 2008; Kicheva et al., 2014; Liem et al., 2000; McMahon et al., 1998; Mizutani et al., 2006). *Ex vivo* assays of neural progenitors indicate that modulating BMP signaling alters the response to a fixed dose of Shh (Liem et al., 2000; Mizutani et al., 2006), and in mouse embryos lacking the BMP inhibitor noggin there is a loss of ventral cell fates despite normal production of Shh protein (McMahon et al., 1998). Likewise, Wnt signaling also inhibits the ventral target genes to promote dorsal identities (Alvarez-Medina et al., 2008). Thus, in both tissues, pattern formation appears to depend on the integration of signaling activities emanating from opposite poles.

A consequence of cross-talk between the anti-parallel gradients is that it results in partial redundancy between the patterning cues. This could contribute to the absence of a strict correlation between morphogen levels and target gene boundaries and the establishment of pattern in embryos in which a gradient has been flattened or removed. For example, although the position of some gap genes is shifted in embryos in which the Bcd gradient has been flattened, well-defined gene expression boundaries continue to form in the correct spatial order (Chen et al., 2012; Ochoa-Espinosa et al., 2009). Perhaps, in these embryos other asymmetric activities that provide polarized inputs into the gap network are revealed (Liu et al., 2013; Löhr et al., 2009). Alternatively, the residual, albeit shallow, gradient observed in the ‘flattened Bcd’ embryos might contribute to the persistence of pattern. Similarly, in the neural tube of mouse embryos lacking Shh and Gli3 (Litingtung and Chiang, 2000; Persson et al., 2002), the signals emanating from the dorsal pole of the neural tube might account for the remaining spatial pattern of ventral pd-TFs (Liem et al., 2000; Mizutani et al., 2006). In this view, the dorsal signals provide differential input into the pd-TFs expressed dorsally and, by repressing ventral pd-TFs, set up the patterns of gene expression.

It is notable in embryos lacking Shh and Gli3 that ventral pattern appears less precise than normal (Litingtung and Chiang, 2000; Persson et al., 2002). Indeed, theoretical analyses indicate that one advantage of the integration of anti-parallel morphogen gradients is that it provides a more accurate way to obtain positional information (Howard and ten Wolde, 2005; McHale et al., 2006; Morishita and Iwasa, 2009; Srinivasan et al., 2014). Using opposing gradients to measure position relative to the two poles of the tissue would allow quantitative adjustments in the formation of pattern, allowing it to scale to the size of the tissue (Howard and ten Wolde, 2005; McHale et al., 2006). In this way, the pattern in a larger individual would be stretched to fit the tissue and vice versa. Alternatively, studies of *Drosophila* strains selected for differences in embryo size show that larger embryos contain consistently higher levels of *bcd* mRNA than smaller embryos (Cheung et al., 2011, 2014). However, a cause-and-effect relationship between amounts of *bcd* RNA and embryo size has not yet been established.

The presence of anti-parallel gradients can also improve the accuracy of patterning by averaging fluctuations in the levels of each morphogen associated with the inherently noisy processes of gradient formation. Molecularly, these mechanisms can be implemented in several ways. For example, one signal could control the expression of components of the transduction pathway of the opposing signal. This might be relevant in the neural tube, where the Shh signaling effector Gli3 appears to be regulated by Wnt activity (Alvarez-Medina et al., 2008). Hence, by acting as a transcriptional repressor of Shh target genes, Gli3 could restrict ventral progenitor specification. Alternatively, mutual repression between pd-TFs that are induced by the opposing gradients also provides a mechanism to increase the precision of boundaries and scale the pattern to embryo size (Manu et al., 2009a,b; Sokolowski et al., 2012; Surkova et al., 2013). In this respect, computational simulations indicate that, in the *Drosophila* blastoderm, the diffusion of gap proteins between nuclei, which is permitted by the lack of cytoplasmic membranes, assists the repair of any errors in patterning while still allowing the rapid generation of sharp boundaries (Tkačik et al., 2015). More complex mechanisms that involve feedback and ‘shuttling’ of morphogen ligands by secreted inhibitors have also been identified in some morphogen-patterned tissues (for a review see Shilo et al., 2013). Further investigations will be necessary to gain a better molecular understanding of the various mechanisms and the contributions that they make in each tissue.

## Conclusions and perspectives

The combined experimental and computational modeling approaches described here build upon the morphogen and positional information concepts developed over the last half century, but support revisions to the theory. Central to this are three ideas. First, gradients establish tissue polarity, but do not pattern tissues via strict concentration thresholds. Thus, there is no strict correspondence between specific threshold concentrations of a morphogen and the position of a gene expression boundary. Second, pattern formation is achieved through transcriptional networks comprising gradient effectors, uniformly expressed factors and pd-TFs that respond to and refine the graded inputs. These transcriptional activities are interpreted by modular regulatory elements containing clusters of binding sites for the network of factors. Third, the integration of gradient-induced polarity with the transcription network produces a dynamical system that refines and positions gene expression boundaries along the patterning axis. Together, this means that positional information is not a static measure but a process that arises from the dynamics of interactions within the network.

These principles might apply to other morphogen-patterned systems. An example in the *Drosophila* embryo is the Dorsal (Dl) morphogen, which is crucial for establishing target gene expression patterns at specific positions along the dorsal-ventral (DV) axis (Roth et al., 1989). There is good evidence that Dl target genes are differentially sensitive to Dl concentrations, but, at the level of the CREs associated with Dl target genes, activation mechanisms are combinatorial, with multiple proteins (Twist and Zld) involved in refining the apparent sensitivities of individual target genes (Foo et al., 2014; Hong et al., 2008b; Jiang and Levine, 1993). There is also support for the idea that the binding of repressors to target gene CREs is important for boundary positioning (Crocker and Erives, 2013; Ozdemir et al., 2014), which echoes the interplay between activators and repressors along the *Drosophila* AP axis and in the vertebrate neural tube.

A major consequence of this view of morphogen patterning is that there is no mechanistic difference between spatial and temporal patterning: both spatial gradients and temporal changes in morphogen input can produce similar gene expression patterns. This might explain apparently conflicting observations that have argued against the importance of the long-range spread of a morphogen ligand in some tissues (see Box 3). Moreover, boundary precision and size scaling are built into the system. The system is robust to fluctuations in the morphogen signal and provides an effective memory when morphogen signal declines, which offers an explanation for the striking ‘canalization’ of pattern formation in many developing tissues.

**Box 3. Other morphogen-based patterning systems: the case of Wingless (Wg)**

The Wnt family member Wg has been implicated in patterning the DV axis of the *Drosophila* wing disc (Campbell and Tomlinson, 1999; Jaźwińska et al., 1999; Minami et al., 1999; Neumann and Cohen, 1997; Zecca et al., 1996). Wg is secreted from the DV boundary at the center of the wing disc, forming a long-range gradient, and experimental evidence suggests that cells distant from the boundary respond directly to Wg. Nevertheless, recent studies revealed that a membrane-tethered version of Wg, which is not released from cells, is able to pattern the DV axis almost as well as secreted Wg (Alexandre et al., 2014). This challenges the requirement for a spatial gradient of Wg. One possible explanation is that Wg expression in the wing disc is dynamic. At early developmental stages, Wg is expressed throughout the disc but, over time, the expression of Wg becomes restricted to the DV boundary (Alexandre et al., 2014). Thus, cells furthest from the DV boundary at the lateral margins of the wing are exposed to Wg for only a brief time at early developmental stages, whereas those closer to the DV boundary receive Wg for longer periods of time. If Wg is interpreted by a transcriptional network that operates with similar principles to the blastoderm and neural tube networks, then different durations of Wg signaling will have the same effect as a spatial gradient of Wg. In this view, either a spatial or temporal gradient of Wg (or a combination of both) could direct pattern formation, and assaying the dynamics or outcome of patterning would not distinguish between static gradient and temporal patterning mechanisms.

Consistent with this, unbiased computational analyses and screens for artificial transcriptional circuits capable of producing stripes of gene expression have also identified mechanisms that rely on the dynamics of the network (Cotterell and Sharpe, 2010; François and Siggia, 2010). A systematic survey of morphogen-regulated networks comprising three TFs identified six distinct classes of network design that generated striped gene expression (Cotterell and Sharpe, 2010). Each of these used a different dynamical mechanism to interpret the morphogen but all relied on cross-regulatory interactions between the TFs. Similarly, an *in silico* evolutionary approach to identify transcriptional networks that interpret either static or dynamic morphogen gradients also resulted in cross-regulatory networks, the structures of which were reminiscent of known morphogen interpreting networks (François and Siggia, 2010). Notably, in this study, networks that had evolved to interpret temporal changes in morphogen signaling were also capable of pattern formation when challenged with a static spatial gradient. This emphasizes the importance of network dynamics for understanding pattern formation and supports the idea that the mechanisms identified in the gap gene and neural tube networks represent general principles for morphogen interpretation.

Despite much progress, many questions remain. Elucidating the components and operation of the transcriptional networks continues and, for many tissues, the relative importance of the spatial or temporal component of gradients needs to be determined. How opposing gradients cross-talk and are integrated into networks is poorly understood. New technologies (e.g. CRISPR/Cas9) will permit the manipulation of regulatory sequences in the native locus, which should allow rapid progress in understanding how patterning information is integrated. Alongside these experimental objectives, improved models and simulations will undoubtedly be important and necessitate improved quantification of the components of the systems. This includes not only measuring the number of molecules of key TFs but also measurements of protein-DNA interactions and rates of transcription and translation of target genes. Models that simplify and abstract aspects of a system will help provide an intuitive understanding of its operation, whereas increasingly complex simulations will result in more realistic models and a means to interpret more and diverse forms of data. Together, therefore, our comparison of patterning in the *Drosophila* blastoderm and the vertebrate neural tube suggests a unified framework for morphogen-mediated pattern formation and establishes a research agenda that will likely take us through further revisions of this fascinating problem.
